# Belonging through values: ethical leadership, creativity, and psychological safety with ethical climate as a moderator

**DOI:** 10.3389/fpsyg.2025.1559427

**Published:** 2025-05-02

**Authors:** Muhammad Qasim, Azhar Ali Laghari

**Affiliations:** ^1^Business School, University of International Business and Economics, Beijing, China; ^2^The College of Resources and Environment (Institute of Agricultural Environment and Resources), Shanxi Agricultural University, Jinzhong, China

**Keywords:** ethical leadership, ethical climate, belongingness, psychological safety, value congruence, creativity

## Abstract

**Introduction:**

This study aims to provide conceptual insights on how varying levels of value congruence vary employees’ sense of belongingness within ethically led organizations. The ethical leadership effect was tested directly and indirectly through belongingness and psychological safety on creativity; the ethical climate was considered as a moderator.

**Methods:**

Study 1 data was multi-sourced and was collected from 377 participants at three different time points. Study 2 was scenario-based data collection. 208 employees participated in this study. The process and Hayes techniques were used for SPSS.

**Results:**

Findings were that ethical leadership was a significant factor in influence directly and indirectly through belongingness and psychological safety on creativity; the moderating role of ethical climate was also found significant. Belongingness varies at high and low levels of congruency with the leader.

**Conclusion:**

Findings suggest that ethical leadership is a strong predictor of belongingness and psychological safety that helps employees to be creative. Overall, the working climate, if it is ethical, also improves the impact of ethical leadership. Then, it discusses the theoretical and practical implications of ethical leadership for research and practices.

## Introduction

The impact of ethical leadership on organizational outcomes has become a pivotal focus, especially given its influence on fostering positive work environments and promoting employee engagement (Fischer and Sitkin, 2023). Like once Martin Luther King said “I am not interested in power for power’s sake, but I am interested in power that is moral, that is right and that is good.” Ethical leadership has been shown to create climates that enhance performance and well-being, contributing to overall organizational success (Shafique et al., 2020). In raising with interest in ethical leadership, what consequences it can have on follower behaviors have been recognized, but how does this give a sense of belonging? This need to belong plays a central role in follower behaviors. We thus find it of greater practical implications to study the link between ethical leadership and creativity through belongingness. The previous research focus was pointed toward ethical leadership on follower extra effort (Tu and Lu, 2016), follower-helping behavior (Li et al., 2023), engagement, and commitment (Asif et al., 2019) but has neglected the role of a situational leadership lens through and what conditions these relationships can be built. Owing to individual differences, followers may have different levels of sensitivity to leadership.

The question we seek to address is: Which individuals are most positively influenced by ethical leadership, and how might those who share similar values feel a greater sense of belonging and alignment under such leadership? We aim to explore how ethical leadership can create an environment where individuals feel valued and connected, particularly when they see their leader embodying the same principles they hold dear. Research suggests that ethical leadership promotes accountability and integrity (Brown et al., 2005), yet critical, relational dynamics underpin employees’ behaviors in ethical leadership contexts. For instance, beyond structural incentives or leader attributes, value congruence between employees and leaders—an alignment of beliefs and values—can profoundly shape how employees perceive their belongingness to the organization. Such a sense of belongingness, though underexplored, is instrumental in cultivating psychological safety, which in turn encourages employees to engage in creative behaviors.

Creativity requires an environment where employees feel safe to express new ideas and take risks. Nonetheless, the roles of belongingness and psychological safety as mediators in this process have been considered vital. Understanding how these psychological processes enable ethical leadership to foster creativity is crucial, as creativity fuels innovation and organizational adaptability (Sawyer and Henriksen, 2024). Investigating these mechanisms is essential for organizations seeking to harness ethical leadership not only to improve performance but also to drive innovation (Amabile and Khaire, 2008). By fostering environments that support a sense of belonging and psychological safety, ethical leaders can help unlock the creative potential of employees, making this an important area of study.

Furthermore, it is important to foster a supportive ethical climate and enhance employees’ sense of belonging and psychological safety to maximize the creative potential within organizations (Javed et al., 2018). These insights offer valuable implications for leaders and practitioners aiming to cultivate ethical and innovative workplaces (Den Hartog, 2015). This research studies the relationship between employees’ low and high levels of value congruency and their sense of belonging within an organization. By analyzing insights into organizational ethical beliefs, this study explores how alignment with organizational ethics enhances employees’ feelings of inclusion. For instance, it is commonly assumed that the leader’s values are the “norm” and that employees will naturally align with these values. However, employees have their unique interests and values, which may or may not align with those of their leader. As such, ethical leadership literature has yet to consider examining the match or mismatch between leader and employee values in order to determine follower outcomes. Employees who possess high-value congruency are more likely to experience a sense of belonging when working under ethical leaders. This alignment fosters stronger organizational commitment, as congruence between employees and leaders can deepen workplace attachment and reinforce organizational identity (Ahmad and Umrani, 2019; Qin et al., 2021).

A lack of research examining the specific pathways through which these effects occur (Hendriks et al., 2020) motivates us to test the serial mediation mechanisms involving belongingness and psychological safety. Understanding how these mediating processes can provide deeper insights into the impacts of ethical leadership on creativity, which is essential for organizational innovation and competitiveness (Asif et al., 2019), is crucial.

This article explores the impact of ethical leadership on employees’ sense of belonging and psychological safety and how these factors contribute to their creativity. While ethical leadership is known to foster a positive organizational climate (Brown and Treviño, 2006), the specific psychological processes linking ethical leadership to creativity remain underexplored (Al Halbusi et al., 2023). Additionally, the moderating role of ethical climate and value congruence in this relationship has yet to be thoroughly examined (Hegarty and Moccia, 2018). Addressing these questions offers new insights into how ethical leadership can be strategically capitalized on to unlock greater employee creativity.

Our research makes several important contributions to the literature on ethical leadership and organizational behavior. First, by identifying belongingness and psychological safety as serial mediators, we provide a more nuanced understanding of the pathways through which ethical leadership influences creativity. Second, this study suggests that employees with strong ethical principles may find increased alignment and purpose when their values are mirrored by ethical leadership, ultimately enhancing their sense of belonging and satisfaction at work (Aftab et al., 2023). Third, our investigation into the moderating role of ethical climate offers new insights into how organizational context can enhance the effectiveness of ethical leadership. Finally, our study extends the application of ethical leadership to the domain of creativity, highlighting its potential to drive innovative behaviors in the workplace. These findings have significant implications for both researchers and practitioners seeking to foster ethical and creative work environments.

### Literature review and hypothesis development

Ethical leadership is distinguished by the leader’s demonstration of normatively appropriate behavior, influencing followers through personal conduct and two-way communication (Brown et al., 2005). This leadership style focuses on promoting ethics and integrity, contrasting it with other leadership approaches such as servant, authentic, or transformational leadership (Avolio et al., 1999; Bass and Avolio, 1993). That is why it has been a topic of interest, and leaders play a crucial role in organizational success and establishing ethical standards (Den Hartog, 2015; Grojean et al., 2004). It seems ethical leadership influence on creativity literature is underdeveloped.

Ethical leaders inspire employees to internalize moral values, which motivates them to engage in creative problem-solving (Feng et al., 2018). Miao et al. (2013) stated that ethical leaders empower employees to take initiative, directly linking ethical behavior to innovative outcomes. Ethical leadership creates a ‘safe space’ for experimentation, a critical precursor to creativity (Liu et al., 2020). Ethical leaders are expected to be fair, caring, and trustworthy, setting an example through their actions (Ko et al., 2018). Ethical leadership has been linked to numerous positive outcomes, including enhanced employee performance, trust, and job satisfaction (Ahn et al., 2018; Gerpott et al., 2019; Tu et al., 2019). Furthermore, ethical leadership can reduce negative behaviors, like staff misconduct, by setting a moral example for employees to follow (Mayer et al., 2010).

Theoretical frameworks such as social exchange theory (SET) and social learning theory have been widely used to explain how ethical leadership influences employee behavior (Fan et al., 2021; Xu et al., 2016). According to Bandura and Walters's (1977) social learning theory, employees imitate the behavior of ethical leaders, while Blau (2017) social exchange theory suggests that individuals reciprocate ethical treatment with ethical behavior. These theories provide insight into the mechanisms through which ethical leadership positively impacts the workplace, such as employees’ creative behavior.

Creativity involves generating innovative and valuable ideas related to products, services, and processes (Madjar et al., 2002; Zhou and Shalley, 2003). It seeks innovative approaches and innovative concepts to create new opportunities (Amabile, 1983). Employees pursuing creative tasks often have disagreements with supervisors, making leadership assistance essential for fostering creativity in non-routine work (Amabile and Gryskiewicz, 1987; Cheung and Wong, 2011). Creativity challenges conventional thinking, it encourages calculated risk-taking, constructive conflict, and questioning authority (Baucus et al., 2008). Ethical leaders do not shy away from giving recognition, regulation, punishment, and judging without personal bias (Feng et al., 2018).

Ethical leadership is recognized for its ability to foster environments that encourage openness and innovation, where employees feel secure expressing novel ideas and take calculated risks without fear of negative repercussions (Edmondson and Lei, 2014), which are essential to enhance employee creativity. This empowerment to think creatively, coupled with a clear moral framework, propels employees to move away from conventional boundaries and experiment with new ways of solving problems (Amabile, 1983). As such, ethical leadership stimulates the creativity of employees, encouraging them to create unique and valuable solutions within the workplace. So we hypothesize that:

*H1:* Ethical leadership has a positive effect on creativity.

### Belongingness, psychological safety, and creativity

Human beings are inherently social, driven by a need to form relationships that support adaptation and survival (Baumeister and Leary, 2017; Hogg, 2009). This “need to belong” reflects the fundamental desire to form close bonds, and failure to satisfy this need can have significant implications for both mental and physical health (Pillow et al., 2015). However, simply having relationships is not enough; individuals seek a deeper sense of belongingness, which involves feeling a fit and acceptance within their social environment (Allen et al., 2022). Literature lacks an understanding of how this need can be fulfilled at the workplace with the assistance of ethical leadership.

Belongingness extends beyond surface-level relationships. It’s about feeling integral to a system or group that provides symbolic meaning and continuity (Leary and Gabriel, 2022). This need for social connection can be linked to various psychological frameworks, such as sociometer theory, which explains the link between relational value and self-esteem (Leary and Baumeister, 2000). Belongingness thus serves as a metric for how individuals perceive their value and place in the social world, offering both stability and a shared identity (Baumeister and Leary, 2017).

Moreover, strong social bonds and a sense of belonging can help individuals navigate uncertainties and give life a broader meaning (Hogg, 2009; Lambert et al., 2013). The ability to feel valued within a group, whether it be personal or organizational, plays a crucial role in personal well-being and collective achievement (Hagerty et al., 1992). Ethical leadership “walk the talk” and always into “what is the right thing?” It is not just maintaining ethicality, it also delegates responsibility their followers, which makes them feel respected and valued (Tu et al., 2019).

In today’s dynamic business world, organizations increasingly rely on employees to improve processes and practices by voicing new ideas, collaborating, and experimenting with innovative methods (Edmondson, 1999; Nembhard and Edmondson, 2011). These activities can pose risks to employees, such as challenging established norms or facing disapproval from colleagues (Tyagi et al., 2017). Employees may hesitate to contribute to learning processes, which can slow both individual and organizational learning (Padmantyo, 2023; Lisetyaningrum and Padmantyo, 2024). Creating a psychologically safe work environment, where employees feel safe to voice ideas, seek feedback, and take risks, is crucial to overcoming these barriers (Edmondson, 1999).

Recent studies emphasize that psychological safety is a key trait of high-performing teams, particularly in safety-critical industries such as healthcare and aviation, where it has been shown to reduce errors and enhance safety (Agarwal and Farndale, 2017; Leroy et al., 2012). Moreover, psychological safety increases team and individual learning across various organizational contexts (Liu and Ge, 2020; Ortega et al., 2014). In this study, we explore psychological safety to better understand its role in fostering effective learning and performance in the workplace.

Employees in the workplace want their managers to display exemplary ethical behavior (Ko et al., 2018). Employers and employees equally want them/themselves to be more fully integrated into the company (Jasintha, 2022). Ethical leadership enhances organizational identification, related to belongingness (Zhang et al., 2023). Therefore this helps them to perceive being embedded in the organization. Belongingness through inclusion and acceptance may facilitate the freedom to express oneself at work. Zimmerman et al. (2025), also suggested that employees have desires to express their authentic selves at work and feel accepted for it. Leaders help them seek out opportunities and support and encourage followers to feel safe in fulfilling rules (Feng et al., 2018). Safety is important because creativity needs to be risky sometimes, ethical leadership makes this possible with fairness and respect for employees. In such an environment, employees may be valued and connected. These feelings can develop psychologically safer thoughts and may be willing to engage in risky activities. It is beneficial for employees to not be worried about failures, which can facilitate creativity.

It is essential to understand how ethical leadership influences employee creativity. Belonging, the fundamental human need to be part of a society, ensures employees feel valued and connected to their organization (Baumeister and Leary, 2017). When employees feel a sense of belonging, they are more likely to engage with others, share ideas, and collaborate effectively. Furthermore, psychological safety allows employees to express their thoughts and ideas without the fear of being judged or ridiculed (Edmondson, 1999). Together, these factors create an atmosphere where creativity can flourish. In an ethically-led organization, leaders promote this environment by promoting inclusivity and ensuring fair treatment, which enhances both the sense of belonging and psychological safety, resulting in heightened creativity. Therefore, belonging and psychological safety are essential components of ethical leadership, which leads to creative outcomes.

*H2:* Belongingness and psychological safety serially mediate the positive effect of ethical leadership on creativity.

### The moderating role of ethical climate

The ethical climate refers to “the prevailing perceptions of typical organizational practices and procedures that have ethical content” (Victor and Cullen, 1988, p. 101). It shares similarities with moral norms, which guide decisions on right and wrong within organizations (Ellemers et al., 2008; Pagliaro et al., 2011). Different organizations cultivate unique subcultures that shape how individuals interact and behave. Research on ethical climate has shown that workplaces with strong ethical climates are linked to higher levels of employee ethics (Verbeke et al., 1996; Treviño et al., 1998) and positive workplace attitudes, such as job satisfaction and loyalty (Newman et al., 2017).

The multidimensional nature of ethical climate has been widely studied. While some view ethical climate as a singular concept (Arnaud, 2010), the more prevalent perspective is that it consists of several interrelated dimensions (Victor and Cullen, 1988; DeConinck, 2011). Victor and Cullen (1987, 1988) proposed a theoretical typology of ethical climates, including nine categories based on two dimensions: the criteria for ethical judgments (egoism, kindness, principles) and the locus of analysis (individual, local, cosmopolitan). This typology has influenced further categorization of ethical climates, distinguishing between climates of self-interest, which emphasize individualistic approaches, and friendship, which emphasize collective approaches to ethical issues (Cullen et al., 1993). These studies also suggested that there is a need to move beyond categorization and observe how it can influence employees at the workplace.

Ethical climate is conceptualized as an organizational-level construct that reflects employees’ shared perceptions of ethical norms and expected behaviors within the organization (Victor and Cullen, 1988). While ethical leadership can influence ethical climate over time, the two constructs remain distinct in that ethical climate emerges from broader organizational systems and shared perceptions beyond any single leader’s influence. Leadership does not operate in a vacuum but is embedded within a larger organizational and cultural context that can either enhance or constrain its effectiveness (Treviño et al., 1998). The extent to which cultural and organizational systems provide ethical conduct determines how strongly ethical leadership influences follower behavior. Porter and McLaughlin (2006) critique the notion of leadership as an isolated phenomenon, arguing that leaders function within complex systems that shape their ability to influence. Leaders face constraints from certain factors, such as structural goals, policies, and competing priorities (Lord et al., 2001). Ethical culture, as a contextual factor, interacts with ethical leadership by shaping how employees interpret and respond to a leader’s ethical behaviors (Argyropoulou and Lintzerakou, 2025). In organizations with a strong ethical climate, ethical leadership is likely to have a stronger influence, as employees perceive ethical norms as both expected and reinforced at multiple levels. Conversely, in weaker ethical climates, the impact of ethical leadership may be diluted due to conflicting organizational signals. This interaction underscores the fluid and context-sensitive nature of leadership (Lord et al., 2001), where multiple layers of context—ranging from organizational policies to broader cultural values—play a role in shaping leader-follower dynamics (Argyropoulou and Lintzerakou, 2025).

The mechanism by which ethical climate moderates ethical leadership outcomes remains underexplored. This study examines the relationship between these ethical climates and employee behavior, particularly creativity, which encompasses certain discretionary actions beneficial to the organization (Chen and Hou, 2016). Ethical climate encompasses the shared perceptions of what is acceptable ethical behavior within an organization (Victor and Cullen, 1988). When an ethical climate is high, it reinforces the values espoused by ethical leaders, ensuring that these values are not just demonstrated by leadership but embedded in the organization’s cultural fabric. This alignment between leadership and organizational values enhances the impact of ethical leadership on employees’ sense of belonging, as employees perceive the consistency in ethical standards throughout the organization. In contrast, a low ethical climate may undermine the efforts of ethical leaders, resulting in a weakening of their influence on employees’ sense of belonging. Thus, the ethical climate promotes the relationship between ethical leadership and belonging, enhancing it in environments with a high ethical climate.

*H3:* The relationship between ethical leadership and employee belongingness is moderated by ethical climate, such that the relationship is stronger when the ethical climate is high than low.

Ethical climate also plays a crucial role in altering the indirect effect of ethical leadership on creativity through belonging and psychological safety. In organizations with a strong ethical climate, the influence of ethical leadership on belongingness and psychological safety is enhanced, as employees experience a cohesive ethical framework that guides behavior and decision-making. Therefore, we argue that this heightened sense of ethical consistency enables employees to feel more secure in expressing creative ideas, as they are reassured by both their leaders and the broader organization. In such environments, the indirect path from ethical leadership to creativity, mediated by belongingness and psychological safety, becomes even more powerful. Conversely, in organizations with a weak ethical climate, the disparity between leadership and organizational practices may decrease the effects of ethical leadership, leading to a weaker indirect effects on creativity. Therefore, the ethical climate regulates the entire serial mediation process, enhancing the pathway from ethical leadership to creativity when the ethical climate is robust.

*H4:* Ethical climate moderates the indirect effect of ethical leadership on creativity via belongingness, such that the indirect effect is stronger when the ethical climate is high rather than low.

### Moderating role of value congruence

LMX theory emphasizes that high-quality interactions between leaders and employees are built on mutual trust, respect, and a sense of obligation, which in turn foster stronger relational bonds (Graen and Uhl-Bien, 1995). In this context, value congruence plays a pivotal role in improving the quality of leader-member relationships. Specifically, when leaders and employees share similar ethical values, they experience a deeper sense of connection and mutual understanding, which bolsters employees’ trust in their leaders and enhances their identification with the organization (Dey et al., 2022). This alignment of values magnifies the benefits of ethical leadership by signaling to employees that their values are respected and shared by their leader, thus fostering a heightened sense of belonging.

Belongingness, as the inherent need to feel accepted and valued within a group, is essential for cultivating psychological safety and stimulating creativity in organizational settings (Aboud et al., 2023). Ethical leadership provides a solid foundation for belongingness by fostering an inclusive and supportive work environment (Walumbwa et al., 2011). However, the degree to which employees feel a sense of belonging under ethical leadership is likely influenced by the extent to which their values align with those of their leader. Studies suggest that value congruence strengthens relational bonds by minimizing value-based conflicts and enhancing mutual understanding (Qu et al., 2019). Therefore, employees who perceive greater value congruence with their leader are more inclined to adopt their leader’s ethical standards, feel a stronger sense of acceptance, and experience deeper belongingness.

Conversely, when value congruence is low, the positive effects of ethical leadership may be weakened, as conflicting values can lead to relational strain and reduce employees’ trust in their leader’s ethical direction (Ilies et al., 2007). This highlights that value congruence is not just an ancillary factor but a crucial moderator that enhances the relational influence of ethical leadership on belongingness. Based on this reasoning, the following hypothesis is proposed:

*H5a:* The relationship between ethical leadership and employees’ sense of belongingness is moderated by value congruence, such that the relationship is stronger when value congruence is high.

*H5b:* Value congruence moderates the indirect effect of ethical leadership on creativity via belongingness, and the indirect effect is stronger when the value congruence is high rather than low.

## Methods

### Research setting

**Figure 1:**
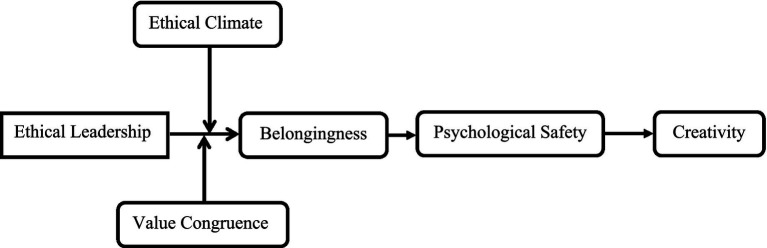
Conceptual model.

This study was conducted within the financial sector of Pakistan, specifically focusing on banks and credit unions. Previous leadership research has largely concentrated on Western contexts, which may not apply to cultures with differing societal norms (Brown and Treviño, 2006). Pakistan, a country with a collectivistic cultural orientation, emphasizes interdependence and the perception of individuals as integral parts of a broader society. This cultural context is particularly relevant for examining ethical leadership, as employees in such environments may be more inclined to work for the benefit of others and align with the ethical standards set by their leaders. The highly competitive nature of the Pakistan financial sector, including credit unions and banks, makes it an ideal setting for exploring leadership behaviors that can influence employees behavior and outcomes (Figure 1).

### Population

**Figure 2:**
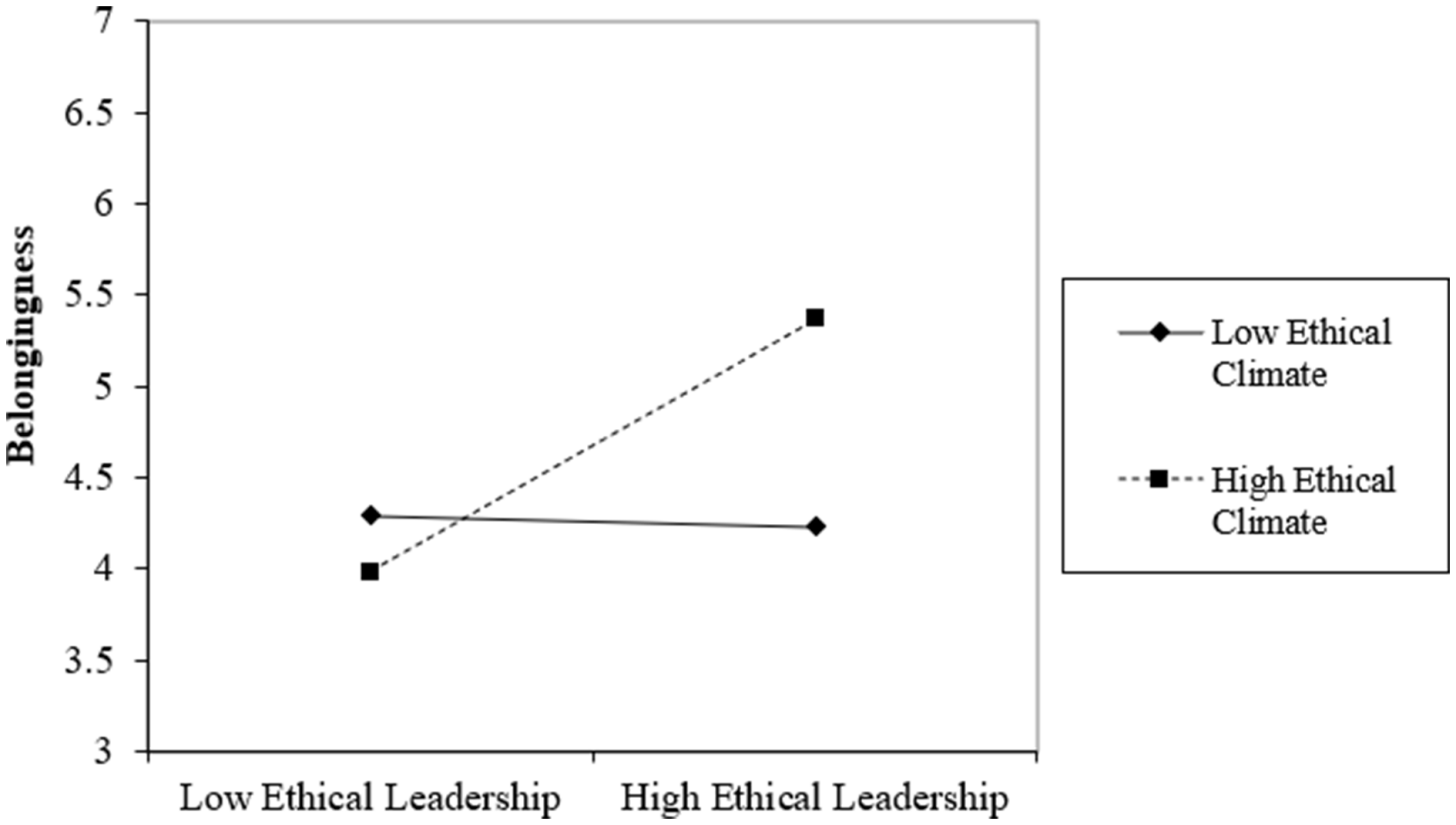
Moderation graph.

The population targeted for this research comprised employees from various departments within financial institutions across Pakistan. With assistance from the National Bank of Pakistan, a comprehensive list of participating banking institutions and other financial entities was compiled, ensuring a representative sample from the sector (Figure 2).

### Sample and sampling methods

A probability-based stratified sampling technique was employed in a two-step process to select participants for this study, and to ensure sufficient representation. Stratified sampling is particularly effective for research covering a geographically dispersed population, ensuring that all subgroups within the population are adequately represented. In the first step, strata were created based on the type of financial institution, such as banks and credit unions. In the second step, institutions were randomly selected from a compiled list provided by the National Bank of Pakistan, ensuring proportional representation across the population. Inclusion criteria for participants included full-time employees with at least one year of employment, while exclusion criteria included temporary or contract workers. This method allowed for a distinct yet representative sample from the financial sector, resulting in the generalizability of the findings.

### Data collection methods

Data were collected at three distinct time intervals using carefully designed questionnaires: Time 1 (T1), Time 2 (T2), and Time 3 (T3). Questionnaires were chosen as the primary data collection method due to their efficient collection of data from a large, geographically dispersed sample while ensuring consistency in measuring constructs across participants. Each questionnaire was specifically tailored to identify relevant concepts and variables to understand the relationships between ethical leadership, psychological safety, and creativity within the organizational context. At T1, 485 participants were contacted; they provided information on their demographic characteristics, ethical leadership, and Islamic work ethics. We got 478 responses back; at this stage, we started checking for some incomplete responses and only contacted 471 for T2. During T2, 471 were contacted, and employees were asked to report on additional constructs such as psychological safety, belongingness, and moral identity; in return, we got 436. After cleaning T2, we came up with 413. Finally, at T3 for 413, employees and their immediate supervisors were asked to provide their perspectives through a separate set of questionnaires. Employees reported the ethical climate of the organization and supervisors’ response to their employees’ creativity. In the end, we had 389 responses. The last four digits of their phone number were used as the identifier. Using time-lagged designs and multisource data helps minimize the risk of common method bias and enhances the ability to infer causal relationships.

After checking for missing values, only 377 responses were valid and complete, making a response rate of 77.7%. This significant response rate enhances the reliability and validity of the findings, providing a solid basis for the subsequent analyses.

To determine the adequacy of the sample size, G*Power software (version 3.1.9.4) was used, following the recommendations of Faul et al. (2009). *A priori* power analysis was conducted using a medium effect size (0.15), an *α* level of 0.05, and a power of 0.95. This analysis indicated that a minimum sample size of 129 was sufficient for testing our model. Furthermore, a *post hoc* power analysis with an actual sample size of 377 yielded a power value of 0.99%, exceeding the minimum threshold of 0.80 (Cohen, 1992). We concluded that our sample size was suitable for testing the hypothesized relationships.

To test the hypotheses of the study, we use PROCESS macro (Hayes, 2018), model 6 (serial mediation) and 1 (simple moderation). This model allows us to observe the indirect paths through which ethical leadership impacts creativity. PROCESS macro was used because it provides flexible and user-friendly frameworks for testing moderating and mediating effects.

## Measurements

Responses were captured on a 7-point Likert scale, ranging from 1 (not at all) to 7 (very much so).

## Ethical leadership

Ethical leadership was rated via a 10-item scale from Brown et al. (2005), which is widely recognized for its reliability in measuring ethical leadership behaviors. Participants were advised to rate their immediate supervisors on two key dimensions of ethical leadership: moral person and moral manager. Sample items include “My supervisor listens to what employees have to say” for the moral manager dimension and “My supervisor conducts his/her personal life in an ethical manner” for the moral person dimension. Ethical leadership was self-reported by employees.

## Belongingness

Belongingness at work was rated using a 3-item scale derived from Godard (2001). An example item is, “When at work, I really feel like I belong.” Belongingness was self-reported by employees.

## Psychological safety

To assess psychological safety, we used Edmondson (1999) 7-item scale. A sample item was, “The work I do is very important to me.” Psychological safety was self-reported by employees.

## Creativity

Employee creativity was assessed using the 4-item scale from Farmer et al. (2003), which has been adapted for use in a Chinese context. A sample item is “This employee: Tries new ideas or methods first.” Supervisors responded to employee creativity.

## Ethical climate

Ethical climate was assessed using the scale of Cullen et al. (1993). A sample item is, “Everyone in this organization is willing to seek common solutions at the workplace.” Employee-rated ethical climate.

## Control variables

This study considered several control variables to account for their potential influence on the research findings. Demographic factors, such as age, education level, and job tenure, were checked for. Prior research suggests that older employees may experience higher levels of job exhaustion (Brienza and Bobocel, 2017), which could reduce the positive impact of ethical leadership. Additionally, educational attainment may influence the generalizability of results, as more educated employees might display more favorable outcomes compared to their less educated counterparts. Job tenure was also controlled, as existing literature (Denton and Kleiman, 2001) has demonstrated its potential effects on employee outcomes.

Furthermore, we considered Islamic work ethics (IWE) and moral identity as control variables to account for their potential impact on employees’ creativity, which could influence the effectiveness of ethical leadership. IWE emphasizes dedication, responsibility, and social relations, which can significantly shape employee behavior and attitudes (Qasim et al., 2022). Moral identity, reflecting to what extent being moral is central to an individual’s self-concept, can also play a crucial role in guiding ethical behavior and fostering creativity (Vitell et al., 2009). Given that the data was collected from Pakistan, a predominantly Muslim country, it is essential to consider these cultural and ethical dimensions, as they might influence work ethics and, consequently, employee creativity (Alqhaiwi et al., 2024; Usman et al., 2015).

## Confirmatory factor analysis and common method bias

In above Table 1, the model fitness indices indicate that the Main 5-Factor Model demonstrates the best fit to the data. With a *χ*^2^/df ratio of 2.11, which is within the acceptable range (≤ 3), and fit indices such as CFI (0.91), TLI (0.90), IFI (0.91), and RMSEA (0.05), this model shows a better model fitness. While we tested other possible combinations as well, compared with the alternate Model 3 (2 factors: Belongingness + Psychological safety + Ethical climate and ethical leadership combined) and alternate Model 4 (1 factor: all constructs combined) show significantly poor fit, with *χ*^2^/df ratios of 4.82 and 7.11, respectively, and fit indices (CFI, TLI, IFI ≤ 0.70; RMSEA ≥ 0.10).

**Table 1:** Model fitness.

Model	*χ* ^2^	Df	*χ*^2^ / df	CFI	TLI	IFI	RMSEA
**Main 5 Factors Model**	1799.5	850	2.11	0.91	0.90	0.91	0.05
**Alternate Model 1:** 4 Factors combine (BE+PS)	2031.6	854	2.37	0.89	0.88	0.89	0.06
**Alternate Model 2:** 3 Factors combine (BE+PS + EL)	3169.9	857	3.69	0.79	0.78	0.79	0.08
**Alternate Model 3:** 2 Factors combine (BE+PS + EL + EC)	4144.6	859	4.82	0.70	0.68	0.70	0.10
**Alternate Model 4**: One Factor Model	6122.3	861	7.11	0.52	0.50	0.52	0.12
Common method variance	6822.9	860	7.93	0.50	0.48	0.51	0.14

BE, belongingness; PS, psychological safety; EL, ethical leadership; EC, ethical climate.

We used time-lagged approaches and multiple source data to reduce the likelihood of common method variance (CMV) (Podsakoff et al., 2012). In our confirmatory factor analysis (CFA), we use the unmeasured latent method construct (ULMC) methodology to verify for CMV (Williams and McGonagle, 2016). We particularly implemented a single-method latent variable into the previously established main model. When we compare our results to the original model, we discovered that including the ULMC made no significant difference in the results. CMV values were far worsen then the main model. As a result, these data suggest that CMV did not significantly damage the reproducibility of our study.

## Assessment of measurement model

In Table 2, AVE values for all constructs exceed the threshold of 0.50, indicating strong convergent validity. The square root of the AVE for each construct (shown in parentheses on the diagonal) is greater than the correlations between that construct and all other constructs. This satisfies the Fornell-Larcker Criterion, confirming discriminant validity. The HTMT values (below the diagonal) represent the ratio of between-construct correlations to within-construct correlations. All HTMT values are well below the conservative threshold of 0.85, further supporting discriminant validity.

**Table 2:** Average variance extracted (AVE), Fornell-Larcker Criterion, and Heterotrait-Monotrait ratio (HTMT).

Order	Variables	AVE	1	2	3	4	5
1	Belongingness	0.578	(0.760)				
2	Creativity	0.543	0.668	(0.737)			
3	Ethical climate	0.607	0.229	0.225	(0.779)		
4	Ethical leadership	0.590	0.381	0.371	0.259	(0.768)	
5	Psychological safety	0.644	0.516	0.464	0.355	0.522	(0.803)

Fornell-Larcker Criterion values shown in parentheses on the diagonal.

## Results

The study examines the relationships among ethical leadership, ethical climate, belongingness, psychological safety, and creativity. Ethical leadership was significantly positively correlated with belongingness (*r* = 0.331, *p* < 0.01), psychological safety (*r* = 0.480, *p* < 0.01), and creativity (*r* = 0.326, *p* < 0.01) (see Table 3). Ethical climate was also positively associated with belongingness (*r* = 0.207, *p* < 0.01), psychological safety (*r* = 0.326, *p* < 0.01), and creativity (*r* = 0.201, *p* < 0.01). Regarding the control variables Islamic Work Ethics (IWE), IWE was positively correlated with creativity (*r* = 0.213, *p* < 0.01). A weaker positive relationship was seen between IWE and belongingness (*r* = 0.130, *p* < 0.05). Moral identity was significantly associated with psychological safety (*r* = 0.205, *p* < 0.01).

**Table 3:** Descriptive statistics, correlations, and reliability values.

Variables	Mean	S. D	1	2	3	4	5
Ethical leadership	5.19	0.93	(0.91)				
Ethical climate	5.17	0.93	0.246^**^	(0.73)			
Belongingness	4.94	1.18	0.331^**^	0.207^**^	(0.92)		
Psychological safety	5.34	1.03	0.480^**^	0.326^**^	0.441^**^	(0.94)	
Creativity	4.83	1.19	0.326^**^	0.201^**^	0.544^**^	0.403^**^	(0.82)
IWE	4.07	0.92	−0.034	0.203^**^	0.130^*^	0.089	0.213
Moral identity	4.95	0.97	0.164^**^	0.233^**^	0.059	0.205^**^	0.007
Age	39.7	9.41	−0.031	0.040	0.083	0.033	0.017
Gender	-	-	−0.081	−0.010	−0.081	−0.07	−0.103^*^
Position	-	-	0.072	0.066	0.069	0.089	0.078
Education	-	-	0.027	0.046	−0.122^*^	−0.025	−0.08
Tenure	-	-	−0.070	0.010	0.013	0.070	0.03

*N* = 377; p** < 0.01; p* < 0.05; IWE, Islamic work ethics “Reliability values are presented in parentheses”.

## Hypothesis testing

For regression, models 6 (serial mediation) and 1 (moderation) were used. Results for our control variables showed (refer to Table 4) that IWE had significant positive effects on belongingness (*B* = 0.18, *p* < 0.01) and creativity (*B* = 0.20, *p* < 0.01). However, no significant effect was found for IWE on psychological safety. For Moral Identity, there was a significant positive relationship with belongingness (*B* = 0.14, *p* < 0.01). Regarding the study variables, Ethical Leadership had a strong positive effect on belongingness (*B* = 0.42, *p* < 0.01), psychological safety (*B* = 0.40, *p* < 0.01), and creativity (*B* = 0.17, *p* < 0.01).

**Table 4:** Regression analysis.

Predictors	Belongingness			Psychological safety			Creativity			Coefficient	SE	*t*	Coefficient	SE	*t*	Coefficient	SE	*t*
Control variables									
IWE	0.18^**^	0.06	2.92	0.06	0.05	1.27	0.20^**^	0.05	3.59
Moral identity	0.14^**^	0.05	2.90	−0.00	0.04	−0.05	−0.10	0.05	−1.91
Age	0.00	0.01	0.05	0.00	0.01	0.16	−0.01	0.01	−1.26
Gender	−0.15	0.12	−1.30	0.01	0.01	0.31	−0.13	0.10	−1.25
Position	0.03	0.05	0.54	0.04	0.04	1.00	0.02	0.05	0.36
Education	−0.14^*^	0.07	−1.92	0.01	0.06	0.18	−0.10	0.07	−1.46
Tenure	0.02	0.04	0.49	0.07^*^	0.03	2.09	0.03	0.04	0.87
Study variables									
Ethical leadership	0.42^**^	0.06	6.79	0.40^**^	0.05	7.96	0.17^**^	0.06	2.72
Belongingness				0.26^**^	0.04	6.62	0.41^**^	0.05	8.54
Psychological Safety							0.18^**^	0.06	3.04
Ethical climate	0.20^**^	0.07	3.07						
EL x EC	0.19^**^	0.06	3.26						

*N* = 377; p** < 0.01; p* < 0.05; EL, ethical leadership; EC, ethical climate.

Additionally, belongingness significantly predicted psychological safety (*B* = 0.26, *p* < 0.01) and creativity (*B* = 0.41, *p* < 0.01), while psychological safety was also positively related to creativity (*B* = 0.18, *p* < 0.01). The moderation analysis revealed that the interaction between Ethical Leadership and Ethical Climate (EL x EC) was significant in predicting belongingness (*B* = 0.19, *p* < 0.01), indicating that a strong ethical climate enhances the positive effect of ethical leadership on belongingness (Figure 2).

Process and Hayes model 6 mediation analysis demonstrated (Table 5) that Ethical Leadership (EL) positively influences creativity through several mediating pathways. The total indirect effect was significant [*Effect* = 0.265, 95% BootCI (0.153, 0.395)]. Belongingness significantly mediated the relationship between ethical leadership and creativity [*Effect* = 0.173, 95% BootCI (0.097, 0.271)], while the pathway through both belongingness and psychological safety had a smaller yet significant effect [*Effect* = 0.020, 95% BootCI (0.004, 0.053)]. Additionally, psychological safety alone also mediated the relationship between ethical leadership and creativity [*Effect* = 0.072, 95% BootCI (0.021, 0.137)].

**Table 5:** Mediation analysis.

Paths	Effect	Boot SE	Boot LLCI	Boot ULCI
Total	0.265	0.063	0.153	0.395
EL → BE → Creativity	0.173	0.043	0.097	0.271
EL → BE→ PS → Creativity	0.02	0.012	0.004	0.053
EL → PS → Creativity	0.072	0.03	0.021	0.137

*N = 377; EL, ethical leadership; BE, belongingness; PS, psychological safety.*

## Study 1 discussion

Study 1 provided evidence supporting our hypothesis that ethical leadership plays a significant role in enhancing employees’ creativity, both directly and indirectly, through factors such as belongingness and psychological safety. Results indicated that ethical leadership positively influences these mediators, ultimately fostering a creative environment. The findings highlight the critical role of psychological safety and a sense of belonging in enhancing creativity, affirming the social capital and self-determination theories that underscore the influence of these relational and psychological factors on individual performance outcomes.

However, some limitations were identified in Study 1, which we aimed to address in Study 2. First, due to the model’s complexity, it was tested in segments rather than as an integrated whole. Second, despite our effort to obtain accurate measures by using time-lagged data collection from multiple sources, the complexity of the model may limit our ability to draw causal inferences (Kenny and Judd, 2014; Podsakoff et al., 2012).

Study 2 addressed these limitations by manipulating ethical leadership, ethical climate, and value congruence to capture a broader and more objective measure of these constructs. By integrating these manipulations and employing a more controlled, scenario-based methodology, the aim was to isolate and examine the effects of ethical leadership, ethical climate, and value congruence on employee belongingness. This approach was helpful in capturing participants’ sense of belonging under varying leadership conditions, enhancing the capacity to interpret causal mechanisms in alignment with the theoretical framework.

## Study 2 methods

### Procedure

220 participants were recruited from the same credit unions and banks as in the previous study. To show appreciation, small gift boxes were distributed to maintain their motivation level. One of the exclusion criteria was prior participation in Study 1. The experiment began with an introductory note, informing participants that they would read a job-related scenario focusing on leadership behavior, ethical climate, and value congruence. Afterward, they were asked to answer questions based on their judgment of the situation. The scenario content highlighted the leader’s work ethic and the organization’s climate. Ethical leadership was manipulated by describing the leader as either highly “ethical or unethical,” following the approach from van Gils et al. (2015). In Study 1, the ethical climate was assessed by the leaders themselves, which is part of top management. Ethical climate questions are mostly about the top management. However, study 2 manipulates the ethical climate (see Supplementary Appendix A). A 2x2x2 factorial design was employed. Participants were randomly selected for these conditions. After reviewing the scenarios, participants completed questions to check the effectiveness of the manipulation, rated their creativity in handling the work situations, evaluated their belongingness and value congruency, and provided demographic details. Participants also rate the scenario realism questions.

A total of 220 took part; 4 were incomplete, and 8 participants failed the attention check. We ended up with 208 participants. There were 66.3% men and an average age of 38.04 (SD = 9.70) years in the sample. The majority of the participants (47%) hold a bachelor’s degree and (16.3%) a master’s, with only 4% holding a doctorate. The mean for experience was 8.92 (SD = 6.37).

For the realism check, responses for the ethical leadership scenario were 5.83, for the ethical climate scenario 5.65, and for value congruence 4.92, which shows that participants understand the scenario well and can relate to it.

### Manipulations check

We checked the manipulations using an independent sample t-test for ethical leadership and ethical climate to see whether they were successful or not. The results for ethical leadership values were (Mean = 5.99, S. D = 1.01) and for no ethical leadership [*Mean* = 2.62, S. D = 1.79, 95% CI (UL = 2.97, LL = 3.77)], with Cohen’s d 2.31. Checking manipulations for ethical climate, the results for high ethical climate (Mean = 5.90, S. D = 1.25) and low ethical climate [Mean = 2.03, S. D = 1.18, 95% CI (UL = 3.54, LL = 4.21)] showed that manipulations were successful. Cohen’s d was 3.18. Value Congruence manipulation check was also confirmed for high (Mean = 4.72, S. D = 1.41) and low [Mean = 3.02, S. D = 1.27, 95% CI (UL = 1.34, LL = 2.21)] Cohen’s d was 1.27.

### Measures

For the realism of the scenario, they responded on 1–7 from highly unrealistic to highly realistic (Mattila and Cho, 2011). Following van Gils et al. (2015), we used one item “to what extent do you believe that your team leader is an ethical leader?” as manipulation check of the scenario manipulations (1 = not ethical at all, 7 = very ethical).

The 10-item ethical climate scale was developed by (Cullen et al., 1993) to measure the overall ethical environment within an organization. The same scale was used for belongingness, ethical leadership, and creativity. In this study, we deliberately excluded psychological safety as it acts as a following mediator after belongingness in the causal chain. This would prevent a cohesive analysis of the entire model in one step, as seen in Study 1, where testing the model in parts may have obscured key relationships (Bollen, 1989; Kline, 2023). By focusing solely on belongingness as the mediator between ethical leadership and employee creativity while incorporating ethical climate as the moderator, we can test the full model at once. This approach allows for a more robust and comprehensive examination of the interaction effects and overall model dynamics, providing clearer and potentially more significant results (MacCallum and Austin, 2000). While value congruence was measured using the 3-item scale of Cable and DeRue, 2002.

### Hypothesis testing

Table 6 shows that ethical leadership is positively related to belongingness (*r* = 0.42, *p* < 0.01) and creativity (*r* = 0.39, *p* < 0.01). Belongingness also showed a positive correlation with creativity (*r* = 0.51, *p* < 0.01). Ethical climate was positively related to belongingness (*r* = 0.56, *p* < 0.01) and creativity (*r* = 0.46, *p* < 0.01). Value congruence was also positively related to belongingness (*r* = 0.40, *p* < 0.01) and creativity (*r* = 0.20, *p* < 0.01).

**Table 6:** Study 2 Mean, S.D., and correlations analysis.

Order	Variables	Mean	S. D	1	2	3	4	5
1	Ethical leadership	4.24	2.23	-				
2	Ethical climate	3.89	2.28	0.44^**^	(0.89)			
3	Belongingness	3.98	1.92	0.42^**^	0.56^**^	(0.96)		
4	Creativity	3.31	1.87	0.39^**^	0.46^**^	0.51^**^	(0.95)	
5	Value congruence	3.92	1.59	0.31^**^	0.27^**^	0.40^**^	0.20^**^	(0.92)

*N* = 208; ** = *p* < 0.05; * = *P* < 0.01, “Values in parenthesis are the reliability values”.

Note: Ethical leadership has only one item.

Table 7 results shows that ethical leadership has a positive effect on belongingness (*B* = 0.19; *p* < 0.01) and creativity (*B* = 0.18; *p* < 0.01). Belongingness also showed a positive significant effect on creativity (*B* = 0.41; *p* < 0.01). Interaction of ethical leadership and value congruence was seen positive significant (*B* = 0.115; *p* < 0.01), so hypothesis 5 was supported. Moderation of ethical climate (*B* = 0.068; *p* < 0.01) was also confirmed. These results are replicated here in Study 2 as well. In Study 1, hypotheses 1 and 3 were also supported. Hypothesis 2 results were also confirmed here as well, as seen in Table 7 (PROCESS and Hayes model 4), which shows that belongingness mediates the relationship between ethical leadership and creativity (*Effect =* 0.180, 95% CI, LL = 0.104, UL = 0.267) (please see Table 8).

**Table 7:** Study 2 direct and moderating effects.

Predictors	Belongingness				Employee creativity				Coefficient	SE	t	P	Coefficient	SE	t	P
Direct effects								
Ethical leadership	0.19	0.05	3.80	0.000	0.18	0.05	3.32	0.001
Belongingness					0.41	0.06	6.59	0.000
Ethical climate	0.31	0.05	6.22	0.000				
Value congruence	0.31	0.07	4.51	0.000				
Moderating effect								
Ethical leadership x Ethical climate	0.068	0.021	3.24	0.001				
Ethical leadership x Value congruence	0.115	0.030	3.82	0.000				
Ethical climate R2-change		0.027						
Value congruence R2-change		0.037						

*N = 208.*

**Table 8:** Study 2 indirect effects.

(95% bias-corrected confidence interval method)				
Indirect effect	Effect	SE	LL	UL
Ethical leadership → belongingness → creativity	0.180	0.042	0.104	0.267

*N = 208; LL, lower limit; UL, upper limit; SE, standard error.*

### Conditional indirect effect of ethical leadership

Table 9 presents significant moderation results, tested using Model 9 of PROCESS by Andrew Hayes. These results support Hypothesis 4 and 5b, indicating that ethical leadership indirectly influences creativity through a sense of belonging, with this relationship being stronger in a high ethical climate. Specifically, the findings confirm that an increase in ethical climate amplifies this effect. For negative values of ethical climate and value congruence, the effect was negatively significant (*Effect* = −0.092, *LL* = −0.171, *UL* = −0.031). In contrast, for a high ethical climate and value congruence, the effect was substantial (*Effect* = 0.238, *LL* = 0.139, *UL* = 0.350). Table 10 depicts the index of moderated mediation results.

**Table 9:** Conditional indirect effect of ethical leadership on creativity at the values of moderators.

Paths	Moderators: ethical climate and value congruence						EC	VC	Effect	S.E	LLCI	ULCI
Belongingness on creativity	−2.89	−1.92	−0.092	0.036	−0.171	−0.031
	−0.144	0.077	0.080	0.024	0.037	0.129
	2.71	1.74	0.238	0.055	0.139	0.350

*N = 208; LL, lower limit; UL, upper limit; SE, standard error.*

EC, ethical climate.

**Table 10:** Index of moderated mediation.

Paths	Index	SE (Boot)	BootLLCI	BootULCI
With EC	0.028	0.009	0.011	0.048
With VC	0.047	0.015	0.022	0.081

EC, ethical climate; VC, value congruence.

## Study 2 discussion

Stud 1 hypothesis was confirmed here as well; we found the conditional indirect effect of ethical leadership on creativity as well. Value congruence was tested on the high and low levels to see whether employees’ sense of belongingness can be varied or not, which confirmed that it varies between high and low levels of value congruence. Findings underscore that employees with high congruence experience a stronger sense of belonging in organizations led by ethical leaders. Ethical leadership cultivates a culture of trust, respect, and integrity, which resonates with the values of morally conscious employees (Mayer et al., 2012; Peng and Kim, 2020). Such leaders actively promote inclusivity and reinforce ethical standards, which encourages employees to identify with the organizational values and feel a greater connection to their workplace. As a result, employees with high congruence are more likely to perceive themselves as integral members of the organization, enhancing their sense of belonging and commitment (Bojanowska and Piotrowski, 2020; Edwards and Cable, 2009). Conversely, employees with lower congruence may struggle to relate to the ethical framework established by their leaders, leading to feelings of disconnection and reduced engagement with the organizational culture (Van Knippenberg et al., 2004). This misalignment diminishes their sense of belonging, ultimately affecting their job satisfaction and loyalty. Therefore, the congruence between ethical leadership and the values of employees is crucial for fostering a profound sense of belonging.

## General discussion

The main objective of this study was to gain a better understanding of ethical leadership in regulating creativity, through Belongingness and psychological safety. Even though the antecedents and outcomes of ethical leadership has been studied, but only few studies focused on the ethical leadership and creativity (e.g., Asif et al., 2019; Chen and Hou, 2016). Specifically, considering the crucial role of belonging was missing. This study considers belongingness and psychological safety as a serial mediator for employees’ creativity. The findings of Study 1 demonstrate that ethical leadership plays a crucial role in shaping a work environment characterized by belongingness, psychological safety, and creativity. These findings were confirmed in study 2 as well. Similarly, previous research emphasizes that leaders who exhibit fairness, integrity, and care contribute to fostering an inclusive environment where employees feel valued and connected (Nishii, 2013). Ethical leadership fosters a sense of belonging by addressing employees’ emotional needs and facilitating shared decision-making, which can enhances organizational cohesion and commitment. This research differs significantly from prior studies by introducing a serial mediation pathway (belongingness → psychological safety) and positioning ethical climate as a moderator rather than a predictor of trust or compliance (Mayer et al., 2010). Additionally, it uncovers the critical role of value congruence in fostering belongingness, an idea supported by Stouten et al. (2012), who argue that individuals are more likely to align with leaders whose actions resonate with their personal and societal values.

Furthermore, the study highlights the significant relationship between belongingness and psychological safety, reinforcing the idea that employees who feel included are more likely to take risks and express their ideas without fear of negative consequences (Edmondson and Bransby, 2023; Frazier et al., 2017). This is based on established research on the importance of trust and openness in promoting innovation and employee engagement (Baumeister and Leary, 2017). Ethical leaders foster inclusivity, which builds trust, essential for psychological safety. This safety, in turn, liberates employees to engage in creative risk-taking. This finding emphasizes that ethical leadership’s impact on creativity is not just a result of additiveness, but a result of social belonging and cognitive safety, requiring both social belonging and cognitive safety to fully manifest.

Additionally, our findings suggest that employees who perceive a high level of ethical ties with their supervisors are more likely to experience a profound sense of belonging. Baumeister and Leary (2017) notion of the ‘need to belong,’ which is not just about forming relationships, but about feeling embedded and valued within a society. Such belongingness provides employees with symbolic meaning and a shared identity within the organization, enhancing both personal fulfillment and social integration (Lambert et al., 2013; Hogg, 2009). Belonging is to go beyond simple interpersonal connection; it embodies an individual’s desire to be a part of an organization that supports their identity and values. This deeper connection rooted in ethical congruence between leaders and employees reinforces the individual’s perception of relational value, which is crucial for self-esteem and psychological well-being (Leary and Baumeister, 2000; Hagerty et al., 1992). As stated by Leary and Kelly (2008), belonging is a critical factor for individuals to engage in collaborative and innovative activities. This study exhibited that ethical leadership plays a crucial role in cultivating belonging, which ultimately creates a psychologically safe environment conducive to creativity.

Additionally, the moderating role of ethical climate further emphasizes the importance of organizational context in enhancing the effects of ethical leadership. A strong ethical climate ensures that the ethical behaviors modeled by leaders are reinforced throughout the organization, encouraging open communication and employee voice (Demirtas and Akdogan, 2015; Al Halbusi et al., 2021). This connection between ethical climate and leadership further enhances the understanding of how leadership behaviors contribute to tangible organizational outcomes such as creativity. Value congruence was found to show how employees will feel more belonging if they share values with the leader; the high congruence will make employees feel more inclusive. These findings facilitate a more precise understanding of leadership in organizations. Furthermore, conditional indirect effect for both ethical climate and value congruence is crucial, which further extends piecemeal approaches that were limited to only direct effect or mediation.

This study’s two-study design enhances the robustness and generalization of the findings. Study 1 results were confirmed in Study 2 by using a 2x2x2 factorial design to enhance causal inferences. In Study 1 IWE and moral identity were controlled, this is based on potential cultural and religious influences, providing a more comprehensive understanding of ethical leadership’s effects. Study 2 methodological advancements, as prior studies on ethical leadership and creativity primarily rely on survey-based, cross-sectional designs. Study 2 methods can reduce common method bias and allow for causal interpretations, providing a more empirical basis for understanding potential interactions.

### Theoretical implications

This study makes a significant contribution to the growing body of literature on ethical leadership, particularly by integrating the concepts of belongingness and psychological safety. Unlike prior studies that often viewed ethical leadership in isolation (Nembhard and Edmondson, 2011), this research demonstrates that belongingness serves as a critical mediator between leadership and positive employee outcomes such as creativity and safety. The relationship is not solely mediated by moral identity or psychological safety (e.g., Miao et al., 2013; Al Halbusi et al., 2023). By highlighting these relationships, this study adds a new layer to our understanding of how leaders’ ethical conduct influences individual belongingness and their creativity.

Moreover, this research advances social exchange theory by providing empirical evidence that ethical leadership creates reciprocal relationships built on trust and equity, which ultimately promote creativity and psychological safety (Brown et al., 2005). The study’s findings offer a multi-dimensional view of leadership, where employees’ social and emotional needs are crucial to driving innovation and organizational effectiveness (Kim et al., 2022). This challenges the conventional leadership discourse that prioritizes performance and productivity, emphasizing instead the ethical and emotional dynamics that shape workplace interactions.

This research contributes to the understanding of belongingness as a nuanced concept within organizational psychology. By demonstrating that alignment (congruence) with ethical leadership enhances employees’ intrinsic need for symbolic meaning and continuity, our findings extend Baumeister and Leary (2017) foundational ‘need to belong’ theory into workplace contexts, showing how ethical climates foster stable, identity-affirming spaces. Our study enhances the literature on belongingness by integrating ethical congruence as a mechanism that fulfills individuals’ psychological needs for group continuity and personal significance within organizations. This need for deeper, value-based connections in the workplace underscores the sociometer theory’s perspective that social bonds serve as indicators of self-worth, providing stability and fostering commitment (Lambert et al., 2013; Leary and Baumeister, 2000).

The study also introduces the moderating effect of ethical climate, showing how the broader organizational context can amplify or diminish the impact of ethical leadership on belongingness and creativity (Den Hartog, 2015). This extends the literature on ethical leadership by recognizing the importance of organizational structures in reinforcing ethical practices, creating an integrated framework for understanding leadership’s far-reaching implications. An ethical climate promotes psychological safety by promoting fairness, transparency, and trust. When employees perceive that their organization prioritizes ethical behavior, they feel confident in expressing their ideas, taking risks, and experimenting without fear of negative consequences (Al Halbusi et al., 2021). This psychological safety is a crucial element of creativity, as it encourages employees to think innovatively and share unconventional ideas.

The study extends the contributions further by making highly congruent employees feel more like they belong. When employees feel my values are aligned with the leader’s, those values are very dear to them. These inclusive feelings help them try things creatively. These findings suggest that value congruence is the necessary contingency that can satisfy the need to belong. In such highly strengthened environments, ethical leadership reinforces shared values that also foster mutual respect (Edwards and Cable, 2009). Employees may attribute certain values to be ethical and if they are referring to it, it will have much better effect (Stouten et al., 2012). For instance, when employees perceive that their leader’s values align with their own, they experience less cognitive dissonance and are more likely to internalize the leader’s ethical standards. This alignment fosters a shared purpose, which motivates employees to contribute creatively to organizational goals.

Lastly, by situating these findings within diverse organizational and cultural contexts, this study highlights the universality of ethical leadership principles, suggesting that they are applicable across both developed and underdeveloped economies (Men et al., 2020). This expansion into cross-cultural leadership studies opens new avenues for exploring ethical leadership in non-Western settings, offering valuable insights into its global relevance. The fundamental concepts of this study (ethical leadership, belongingnes, psychological safety, and creativity) are relevant in every field. By controlling IWE and Moral identity, we can minimize the culture-specific factors. This framework can be adopted and tested in other cultural and organizational settings. Ethical leadership and a strong ethical climate extend beyond organizational benefits and have a greater impact on society by shaping ethical norms, fostering a culture of integrity, and promoting responsible business practices. Furthermore, ethical leadership encourages innovation and creativity by fostering a psychologically safe environment where employees feel empowered to take risks and create new ideas. This, in turn, drives socially responsible innovation, a workplace culture rooted in ethics can lead to a wider adoption of ethical decisions.

### Practical implications

From a practical perspective, this study provides several actionable insights for organizations seeking to foster creativity, engagement, and psychological safety in the workplace. First, by emphasizing the role of ethical leadership in promoting a sense of belonging, organizations are encouraged to invest in leadership development programs that not only teach ethical decision-making but also cultivate empathy, inclusiveness, and justice in leadership behaviors (Grigoropoulos, 2020). This approach has the potential to transform workplaces by promoting higher levels of employee engagement and loyalty, which in turn drives innovation and organizational success.

Second, the study demonstrates that belongingness is a key driver of both psychological safety and creativity, meaning that organizations should prioritize fostering an inclusive culture where employees feel connected, valued, and involved (Chen et al., 2023). Practical steps include implementing inclusive decision-making processes, establishing recognition programs, and creating spaces for open communication and employee voice (Shafique et al., 2020). These practices not only enhance employee job satisfaction but also strengthen the organization’s capacity to innovate and solve complex problems.

Additionally, the study highlights the critical role of ethical climate in enhancing the positive effects of ethical leadership, suggesting that organizations must create a culture of integrity that supports ethical behavior at all levels (Grigoropoulos, 2020). This can be achieved by establishing ethical guidelines, ensuring transparency in leadership practices, and holding leaders accountable for fostering a fair and respectful work environment (De Cremer and Van Knippenberg, 2003). These organizational practices help create a harmonious and supportive work environment, where employees feel empowered to share their ideas and engage in collaborative problem-solving.

Organizations can enhance employee belongingness by promoting an ethical climate that aligns with employees’ personal values. This sense of being an integral part of an ethically grounded system can contribute to greater workplace satisfaction and retention, as employees find both symbolic and relational value within the organization. The practical value of ethical leadership lies in its potential to support employees’ inherent need for belonging by creating an environment that offers not only surface-level inclusion but also deeper meaning and continuity. For HR leaders, prioritizing value alignment in recruitment and leadership practices could prove essential in building a cohesive, engaged workforce.

Furthermore, this study offers valuable insights for global organizations operating in diverse cultural contexts. The universal applicability of ethical leadership principles suggests that leaders in multinational organizations can benefit from adopting context-sensitive ethical practices that are consistent with local values while maintaining a strong commitment to global ethical standards (Brown et al., 2005). This is particularly relevant in underdeveloped economies, where ethical leadership can drive both organizational growth and employee development.

In conclusion, this study’s practical implications underscore the transformational potential of ethical leadership in creating psychologically safe, innovative, and inclusive workplaces. By embedding ethical leadership within the broader organizational culture, companies can unlock creative potential, improve employee well-being, and ensure sustainable growth in today’s dynamic and competitive business environments (Nishii, 2013; Roberson, 2006).

### Limitations and future research directions

Without any doubt, this study has some limitations that are beneficial to observe. First, the three-time-lagged, dual-source design, while offering stronger temporal inferences than a cross-sectional approach, still does not fully address causality concerns. A longitudinal or experimental approach could assist in addressing causation more rigorously, allowing for the tracking of changes over a longer period and across various conditions. Because literature lacks whether ethical leadership’s caring effects persist for some time or not. Do supervisors maintain their ethical focus under prolonged pressure, or does their commitment wane? Do employees become used to ethical leadership over the past? The ups and downs in leadership and employee methods can only be observed in a longitudinal study. Future research could build on this by adopting such longitudinal methods to provide clearer insights into cause-and-effect relationships. Stouten et al. (2012) suggested that aligning (or diverging) from broader social norms can contribute to creativity and organizational performance. The contents of the box named ethics might have been completely different. Research can also study that ethical leadership might inspire employees by the leader’s ethical behavior, but also employees feel pressure to meet the standards. How ethical follower will respond to ethical leadership, can also be in an interesting field to study.

Second, the scenario-based nature of our second study, self-rated responses may have introduced biases. Although supervisor ratings are appreciated in organizational research, further studies can look at longitudinal and peer/supervisor-rated sourced data. Future research could complement these subjective ratings with objective measures, such as the experience sampling method, to see variations and understandings of value congruence, belongingness, and willingness to be creative. Future research could examine how varying degrees of moral values alignment between employees and leadership influence feelings of belongingness across diverse organizational settings. Recently, research suggested that moral values might play a crucial role in high congruence (Seggewiss et al., 2019).

## Conclusion

Throughout two studies, we show that leaders’ belief in ethics is a crucial factor in the workplace that enhances a sense of belongingness and safety. People exchange relationships or try to learn and imitate their leaders. We use values to see the congruence between ethical leadership and followers, which also affects a sense of belongingness. We use this as a springboard to get the attention of the researchers toward this understudied phenomenon. While it looks very important how employees think about the ethics in an organization and how they value these conducts, it will be based on their value congruence. Different employees will value these conducts in different ways and can relate to themselves; in the words of Dunford et al. (2015: 319), “Your employees are watching.” Different eyes, different perceptions.

## Data Availability

The raw data supporting the conclusions of this article will be made available by the authors, without undue reservation.

## References

[ref1] Aboud K. Xiongying N. Qasim M. (2023). Impact of ethical leadership on employees' psychological safety and voice behavior; with mediating role of belongingness. Int. J. Sci. Busi. 20, 42–57. doi: 10.58970/IJSB.2055

[ref2] Aftab J. Sarwar H. Kiran A. Qureshi M. I. Ishaq M. I. Ambreen S.. (2023). Ethical leadership, workplace spirituality, and job satisfaction: moderating role of self-efficacy. Int. J. Emerg. Mark. 18, 5880–5899. doi: 10.1108/IJOEM-07-2021-1121

[ref3] Agarwal P. Farndale E. (2017). High-performance work systems and creativity implementation: the role of psychological capital and psychological safety. Hum. Resour. Manag. J. 27, 440–458. doi: 10.1111/1748-8583.12148

[ref4] Ahmad I. Umrani W. A. (2019). The impact of ethical leadership style on job satisfaction: mediating role of perception of green HRM and psychological safety. Leadership Organiz. Dev. J. 40, 534–547. doi: 10.1108/LODJ-12-2018-0461

[ref5] Ahn J. Lee S. Yun S. (2018). Leaders’ core self-evaluation, ethical leadership, and employees’ job performance: the moderating role of employees’ exchange ideology. J. Bus. Ethics 148, 457–470. doi: 10.1007/s10551-016-3030-0

[ref6] Al Halbusi H. Ruiz-Palomino P. Morales-Sánchez R. Abdel Fattah F. A. M. (2021). Managerial ethical leadership, ethical climate and employee ethical behavior: does moral attentiveness matter? Ethics Behav. 31, 604–627. doi: 10.1080/10508422.2021.1937628

[ref7] Al Halbusi H. Ruiz-Palomino P. Williams K. A. (2023). Ethical leadership, subordinates’ moral identity and self-control: two-and three-way interaction effect on subordinates’ ethical behavior. J. Bus. Res. 165:114044. doi: 10.1016/j.jbusres.2023.114044

[ref8] Allen K. A. Gray D. L. Baumeister R. F. Leary M. R. (2022). The need to belong: a deep dive into the origins, implications, and future of a foundational construct. Educ. Psychol. Rev. 34, 1133–1156. doi: 10.1007/s10648-021-09633-6, PMID: 34483627 PMC8405711

[ref9] Alqhaiwi Z. O. Koburtay T. Syed J. (2024). The interplay between Islamic work ethic, unethical pro behaviors, and moral identity internalization: the moderating role of religiosity. J. Bus. Ethics 193, 393–408. doi: 10.1007/s10551-023-05527-5

[ref10] Amabile T. M. (1983). The social psychology of creativity: a componential conceptualization. J. Pers. Soc. Psychol. 45, 357–376. doi: 10.1037/0022-3514.45.2.357

[ref11] Amabile T. M. Gryskiewicz N. D. (1987). Creativity in the R&D laboratory. Creat. Res. J. 1, 237–281.

[ref12] Amabile T. M. Khaire M. (2008). Creativity and the role of the leader. J. Manag. Train. Inst. SAIL Ranchi 36, 48–51. 18822674

[ref13] Argyropoulou E. Lintzerakou E. E. (2025). Contextual factors and their impact on ethical leadership in educational settings. Admin. Sci. 15:23. doi: 10.3390/admsci15010023

[ref14] Arnaud A. (2010). Conceptualizing and measuring ethical work climate: development and validation of the ethical climate index. Bus. Soc. 49, 345–358. doi: 10.1177/0007650310362865

[ref15] Asif M. Qing M. Hwang J. Shi H. (2019). Ethical leadership, affective commitment, work engagement, and creativity: testing a multiple mediation approach. Sustain. For. 11:4489. doi: 10.3390/su11164489

[ref16] Avolio B. J. Bass B. M. Jung D. I. (1999). Re-examining the components of transformational and transactional leadership using the multifactor leadership. J. Occup. Organ. Psychol. 72, 441–462. doi: 10.1348/096317999166789

[ref17] Bandura A. Walters R. H. (1977). Social learning theory, vol. 1. Englewood Cliffs, NJ: Prentice Hall, 141–154.

[ref18] Bass B. M. Avolio B. J. (1993). Transformational leadership and organizational culture. Public Adm. Q. 17, 112–121.

[ref19] Baucus M. S. Norton W. I. Baucus D. A. Human S. E. (2008). Fostering creativity and innovation without encouraging unethical behavior. J. Bus. Ethics 81, 97–115. doi: 10.1007/s10551-007-9483-4

[ref20] Baumeister R. F. Leary M. R. (2017). The need to belong: desire for interpersonal attachments as a fundamental human motivation. Interper. Dev. 117, 57–89. doi: 10.4324/9781351153683-3 7777651

[ref21] Blau P. (2017). Exchange and power in social life. New York, NY: John Wiley.

[ref22] Bojanowska A. Piotrowski K. (2020). Is person-group value congruence always a good thing? Values and well-being among maladjusted teens and their peers. Front. Psychol. 11:2035. doi: 10.3389/fpsyg.2020.02035, PMID: 32982847 PMC7479829

[ref23] Bollen K. A. (1989). Structural equations with latent variables. New York: Wiley.

[ref90001] Brienza J. P. Bobocel D. R. (2017). Employee age alters the effects of justice on emotional exhaustion and organizational deviance. Front. Psychol. 8:479. doi: 10.3389/fpsyg.2017.00479 28428764 PMC5382225

[ref24] Brown M. E. Treviño L. K. (2006). Ethical leadership: a review and future directions. Leadersh. Q. 17, 595–616. doi: 10.1016/j.leaqua.2006.10.004

[ref25] Brown M. E. Treviño L. K. Harrison D. A. (2005). Ethical leadership: a social learning perspective for construct development and testing. Organ. Behav. Hum. Decis. Process. 97, 117–134. doi: 10.1016/j.obhdp.2005.03.002

[ref26] Cable D. M. DeRue D. S. (2002). The convergent and discriminant validity of subjective fit perceptions. J. Appl. Psychol. 87, 875–884. doi: 10.1037/0021-9010.87.5.875, PMID: 12395812

[ref27] Chen A. S. Y. Hou Y. H. (2016). The effects of ethical leadership, voice behavior and climates for innovation on creativity: a moderated mediation examination. Leadersh. Q. 27, 1–13. doi: 10.1016/j.leaqua.2015.10.007

[ref28] Chen H. Liang Q. Feng C. Zhang Y. (2023). Leadership and follower voice: the role of inclusive leadership and group faultlines in promoting collective voice behavior. J. Appl. Behav. Sci. 59, 61–87. doi: 10.1177/00218863211035243

[ref29] Cheung M. F. Y. Wong C. S. (2011). Transformational leadership, leader support, and employee creativity. Leader. Organiz. Dev. J. 32, 656–672. doi: 10.1108/01437731111169988

[ref30] Cohen J. (1992). Statistical power analysis. Curr. Dir. Psychol. Sci. 1, 98–101. doi: 10.1111/1467-8721.ep10768783

[ref31] Cullen J. B. Victor B. Bronson J. W. (1993). The ethical climate questionnaire: an assessment of its development and validity. Psychol. Rep. 73, 667–674. doi: 10.2466/pr0.1993.73.2.667

[ref32] De Cremer D. Van Knippenberg D. (2003). Cooperation as a function of leader self-sacrifice, trust, and identification. Leader. Quart. 14, 85–106. doi: 10.1108/01437730510607853

[ref33] DeConinck J. B. (2011). The effects of ethical climate on organizational identification, supervisory trust, and turnover among salespeople. J. Bus. Res. 64, 617–624. doi: 10.1016/j.jbusres.2010.06.014

[ref34] Demirtas O. Akdogan A. A. (2015). The effect of ethical leadership behavior on ethical climate, turnover intention, and affective commitment. J. Bus. Ethics 130, 59–67. doi: 10.1007/s10551-014-2196-6

[ref35] Den Hartog D. N. (2015). Ethical leadership. Annu. Rev. Organ. Psychol. Organ. Behav. 2, 409–434. doi: 10.1146/annurev-orgpsych-032414-111237

[ref36] Denton D. W. Kleiman L. S. (2001). Job tenure as a moderator of the relationship between autonomy and satisfaction. Appl. HRM Res. 6, 105–114.

[ref37] Dey M. Bhattacharjee S. Mahmood M. Uddin M. A. Biswas S. R. (2022). Ethical leadership for better sustainable performance: role of employee values, behavior and ethical climate. J. Clean. Prod. 337:130527. doi: 10.1016/j.jclepro.2022.130527

[ref38] Dunford B. B. Jackson C. L. Boss A. D. Tay L. Boss R. W. (2015). Be fair, your employees are watching: a relational response model of external third-party justice. Pers. Psychol. 68, 319–352. doi: 10.1111/peps.12081

[ref39] Edmondson A. C. (1999). Psychological safety and learning behavior in work teams. Adm. Sci. Q. 44, 350–383. doi: 10.2307/2666999

[ref40] Edmondson A. C. Bransby D. P. (2023). Psychological safety comes of age: observed themes in an established literature. Annu. Rev. Organ. Psych. Organ. Behav. 10, 55–78. doi: 10.1146/annurev-orgpsych-120920-055217

[ref41] Edmondson A. Lei Z. (2014). Psychological safety: the history, renaissance, and future of an interpersonal construct. Annu. Rev. Organ. Psych. Organ. Behav. 1, 23–43. doi: 10.1146/annurev-orgpsych-031413-091305

[ref42] Edwards J. R. Cable D. M. (2009). The value of value congruence. J. Appl. Psychol. 94, 654–677. doi: 10.1037/a0014891, PMID: 19450005

[ref43] Ellemers N. Pagliaro S. Barreto M. Leach C. W. (2008). Is it better to be moral than smart? The effects of morality and competence on the decision to work at group status improvement. J. Pers. Soc. Psychol. 95, 1397–1410. doi: 10.1037/a0012628, PMID: 19025291

[ref44] Fan X. Li J. Mao Z. E. Lu Z. (2021). Can ethical leadership inspire employee loyalty in hotels in China?-from the perspective of the social exchange theory. J. Hosp. Tour. Manag. 49, 538–547. doi: 10.1016/j.jhtm.2021.11.006

[ref45] Farmer S. M. Tierney P. Kung-McIntyre K. (2003). Employee creativity in Taiwan: an application of role identity theory. Acad. Manag. J. 46, 618–630. doi: 10.2307/30040653

[ref46] Faul F. Erdfelder E. Buchner A. Lang A. G. (2009). Statistical power analyses using G* power 3.1: tests for correlation and regression analyses. Behav. Res. Methods 41, 1149–1160. doi: 10.3758/BRM.41.4.1149, PMID: 19897823

[ref47] Feng J. Zhang Y. Liu X. Zhang L. Han X. (2018). Just the right amount of ethics inspires creativity: a cross-level investigation of ethical leadership, intrinsic motivation, and employee creativity. J. Bus. Ethics 153, 645–658. doi: 10.1007/s10551-016-3297-1

[ref48] Fischer T. Sitkin S. B. (2023). Leadership styles: a comprehensive assessment and way forward. Acad. Manag. Ann. 17, 331–372. doi: 10.5465/annals.2020.0340

[ref49] Frazier M. L. Fainshmidt S. Klinger R. L. Pezeshkan A. Vracheva V. (2017). Psychological safety: a meta-analytic review and extension. Pers. Psychol. 70, 113–165. doi: 10.1111/peps.12183

[ref50] Gerpott F. H. Van Quaquebeke N. Schlamp S. Voelpel S. C. (2019). An identity perspective on ethical leadership to explain organizational citizenship behavior: the interaction between leader group prototypicality and leader ethicality. J. Bus. Ethics 156, 1063–1078. doi: 10.1007/s10551-017-3625-0

[ref51] Godard J. (2001). High performance and the transformation of work? The implications of alternative work practices for the experience and outcomes of work. ILR Rev. 54, 776–805. doi: 10.1177/001979390105400402

[ref52] Graen G. B. Uhl-Bien M. (1995). Relationship-based approach to leadership: development of leader-member exchange (LMX) theory of leadership over 25 years: applying a multi-level multi-domain perspective. Leadersh. Q. 6, 219–247. doi: 10.1016/1048-9843(95)90036-5

[ref53] Grigoropoulos J. E. (2020). How can manifesting leadership skills infused with ethos, empathy, and compassion better prepare students to assume leadership roles? Int. J. Progress. Educ. 16, 54–66. doi: 10.29329/ijpe.2020.228.5

[ref54] Grojean M. W. Resick C. J. Dickson M. W. Smith D. B. (2004). Leaders, values, and organizational climate: examining leadership strategies for establishing an organizational climate regarding ethics. J. Bus. Ethics 55, 223–241. doi: 10.1007/s10551-004-1275-5

[ref55] Hagerty B. M. Lynch-Sauer J. Patusky K. L. Bouwsema M. Collier P. (1992). Sense of belonging: a vital mental health concept. Arch. Psychiatr. Nurs. 6, 172–177. doi: 10.1016/0883-9417(92)90028-H 1622293

[ref56] Hayes A. F. (2018). Partial, conditional, and moderated moderated mediation: quantification, inference, and interpretation. Commun. Monogr. 85, 4–40. doi: 10.1080/03637751.2017.1352100

[ref57] Hegarty N. Moccia S. (2018). Components of ethical leadership and their importance in sustaining organizations over the long term. J. Values Based Leader. 11:7. doi: 10.22543/0733.111.1199

[ref58] Hendriks M. Burger M. Rijsenbilt A. Pleeging E. Commandeur H. (2020). Virtuous leadership: a source of employee well-being and trust. Manag. Res. Rev. 43, 951–970. doi: 10.1108/MRR-07-2019-0326

[ref59] Hogg M. A. (2009). Managing self-uncertainty through group identification. Psychol. Inq. 20, 221–224. doi: 10.1080/10478400903333452

[ref60] Ilies R. Nahrgang J. D. Morgeson F. P. (2007). Leader-member exchange and citizenship behaviors: a meta-analysis. J. Appl. Psychol. 92, 269–277. doi: 10.1037/0021-9010.92.1.269, PMID: 17227168

[ref61] Jasintha N. (2022). Ethical leadership and organisational identification: the mediating effect of psychological contract—evidence from Sri Lankan apparel industry. J. Busi. Stud. 9, 23–36. doi: 10.4038/jbs.v9i2.80

[ref62] Javed B. Rawwas M. Y. Khandai S. Shahid K. Tayyeb H. H. (2018). Ethical leadership, trust in leader and creativity: the mediated mechanism and an interacting effect. J. Manag. Organ. 24, 388–405. doi: 10.1017/jmo.2017.56

[ref63] Kenny D. A. Judd C. M. (2014). Power anomalies in testing mediation. Psychol. Sci. 25, 334–339. doi: 10.1177/0956797613502676, PMID: 24311476

[ref64] Kim S. Jeong S. H. Seo M. H. (2022). Nurses' ethical leadership and related outcome variables: systematic review and meta-analysis. J. Nurs. Manag. 30, 2308–2323. doi: 10.1111/jonm.13726 35761760

[ref65] Kline R. B. (2023). Principles and practice of structural equation modeling. New York, NY: Guilford Publications.

[ref66] Ko C. Ma J. Bartnik R. Haney M. H. Kang M. (2018). Ethical leadership: an integrative review and future research agenda. J. Bus. Ethics 149, 1–18. doi: 10.1080/10508422.2017.1318069

[ref67] Lambert N. M. Stillman T. F. Hicks J. A. Kamble S. Baumeister R. F. Fincham F. D. (2013). To belong is to matter: sense of belonging enhances meaning in life. Personal. Soc. Psychol. Bull. 39, 1418–1427. doi: 10.1177/0146167213499186, PMID: 23950557

[ref68] Leary M. R. Baumeister R. F. (2000). The nature and function of self-esteem: sociometer theory. Adv. Exp. Soc. Psychol. 32, 1–62. doi: 10.1016/S0065-2601(00)80003-9

[ref69] Leary M. Gabriel S. (2022). The relentless pursuit of acceptance and belonging. Adv. Motiv. Sci. 9, 135–178. doi: 10.1016/bs.adms.2021.12.001

[ref70] Leary M. R. Kelly K. M. (2008). Belonging motivation. Handbook Individual Differences Social Behavior. 400–409. New York City, NY: Guilford.

[ref71] Leroy H. Palanski M. E. Simons T. (2012). Authentic leadership and behavioral integrity as drivers of follower commitment and performance. J. Bus. Ethics 107, 255–264. doi: 10.1007/s10551-011-1036-1

[ref72] Li S. Jia R. Seufert J. H. Luo J. Sun R. (2023). You may not reap what you sow: how and when ethical leadership promotes subordinates’ online helping behavior: ethical leadership and online helping behavior. Asia Pac. J. Manag. 40, 1683–1702. doi: 10.1007/s10490-022-09831-y

[ref9001] Lisetyaningrum Padmantyo S. (2024). The influence of knowledge management, organizational learning, and risk taking on organizational performance: positive innovation outcomes as an intervening variable. 949–963. doi: 10.2991/978-94-6463-204-0_78

[ref73] Liu K. Ge Y. (2020). How psychological safety influences employee creativity in China: work engagement as a mediator. Soc. Behav. Personal. Int. J. 48, 1–7. doi: 10.2224/sbp.9211

[ref74] Liu X. Liao H. Derfler-Rozin R. Zheng X. Wee E. X. Qiu F. (2020). In line and out of the box: how ethical leaders help offset the negative effect of morality on creativity. J. Appl. Psychol. 105, 1447–1465. doi: 10.1037/apl0000489 32162952

[ref75] Lord R. G. Brown D. J. Harvey J. L. Hall R. J. (2001). Contextual constraints on prototype generation and their multilevel consequences for leadership perceptions. Leadersh. Q. 12, 311–338. doi: 10.1016/S1048-9843(01)00081-9

[ref76] MacCallum R. C. Austin J. T. (2000). Applications of structural equation modeling in psychological research. Annu. Rev. Psychol. 51, 201–226. doi: 10.1146/annurev.psych.51.1.201, PMID: 10751970

[ref77] Madjar N. Oldham G. R. Pratt M. G. (2002). There's no place like home? The contributions of work and nonwork creativity support to employees' creative performance. Acad. Manag. J. 45, 757–767. doi: 10.2307/3069309

[ref78] Mattila A. S. Cho W. (2011). The role of self-service technologies in restoring justice. J. Bus. Res. 64, 348–355. doi: 10.1016/j.jbusres.2010.02.014

[ref79] Mayer D. M. Aquino K. Greenbaum R. L. Kuenzi M. (2012). Who displays ethical leadership, and why does it matter? An examination of antecedents and consequences of ethical leadership. Acad. Manag. J. 55, 151–171. doi: 10.5465/amj.2008.0276

[ref80] Mayer D. M. Kuenzi M. Greenbaum R. L. (2010). Examining the link between ethical leadership and employee misconduct: the mediating role of ethical climate. J. Bus. Ethics 95, 7–16. doi: 10.1007/s10551-011-0794-0

[ref81] Men C. Fong P. S. Huo W. Zhong J. Jia R. Luo J. (2020). Ethical leadership and knowledge hiding: a moderated mediation model of psychological safety and mastery climate. J. Bus. Ethics 166, 461–472. doi: 10.1007/s10551-018-4027-7

[ref82] Miao Q. Newman A. Yu J. Xu L. (2013). The relationship between ethical leadership and unethical pro-organizational behavior: linear or curvilinear effects? J. Bus. Ethics 116, 641–653. doi: 10.1007/s10551-012-1504-2

[ref83] Nembhard I. M. Edmondson A. C. (2011). Psychological safety: A foundation for speaking up, collaboration, and experimentation in organizations. The oxford handbook of positive organizational scholarship. eds. Cameron K. S. Spreitzer G. M. (New York: Oxford University Press) 490–503.

[ref84] Newman A. Donohue R. Eva N. (2017). Psychological safety: a systematic review of the literature. Hum. Resour. Manag. Rev. 27, 521–535. doi: 10.1016/j.hrmr.2017.01.001

[ref86] Nishii L. H. (2013). The benefits of climate for inclusion for gender-diverse groups. Acad. Manag. J. 56, 1754–1774. doi: 10.5465/amj.2009.0823

[ref87] Ortega A. Van den Bossche P. Sánchez-Manzanares M. Rico R. Gil F. (2014). The influence of change-oriented leadership and psychological safety on team learning in healthcare teams. J. Bus. Psychol. 29, 311–321.

[ref88] Padmantyo S. (2023). “The influence of knowledge management, organizational learning, and risk taking on organizational performance: positive innovation outcomes as an intervening variable” in International conference on economics and business studies (ICOEBS-22-2) (Atlantis Press), 949–963. doi: 10.1007/s10869-013-9315-8

[ref89] Pagliaro S. Ellemers N. Barreto M. (2011). Sharing moral values: anticipated ingroup respect as a determinant of adherence to morality-based (but not competence-based) group norms. Personal. Soc. Psychol. Bull. 37, 1117–1129. doi: 10.1177/0146167211406906, PMID: 21540366

[ref90] Peng A. C. Kim D. (2020). A meta-analytic test of the differential pathways linking ethical leadership to normative conduct. J. Organ. Behav. 41, 348–368. doi: 10.1002/job.2427

[ref91] Pillow D. R. Malone G. P. Hale W. J. (2015). The need to belong and its association with fully satisfying relationships: a tale of two measures. Personal. Individ. Differ. 74, 259–264. doi: 10.1016/j.paid.2014.10.031, PMID: 27134325 PMC4848456

[ref92] Podsakoff P. M. MacKenzie S. B. Podsakoff N. P. (2012). Sources of method bias in social science research and recommendations on how to control it. Annu. Rev. Psychol. 63, 539–569. doi: 10.1146/annurev-psych-120710-100452, PMID: 21838546

[ref93] Porter L. W. McLaughlin G. B. (2006). Leadership and the organizational context: like the weather? Leadersh. Q. 17, 559–576. doi: 10.1016/j.leaqua.2006.10.002

[ref94] Qasim M. Irshad M. Majeed M. Rizvi S. T. H. (2022). Examining impact of Islamic work ethic on task performance: mediating effect of psychological capital and a moderating role of ethical leadership. J. Bus. Ethics 180, 283–295. doi: 10.1007/s10551-021-04916-y

[ref95] Qin X. Liu X. Brown J. A. Zheng X. Owens B. P. (2021). Humility harmonized? Exploring whether and how leader and employee humility (in) congruence influences employee citizenship and deviance behaviors. J. Bus. Ethics 170, 147–165. doi: 10.1007/s10551-019-04250-4

[ref96] Qu Y. E. Dasborough M. T. Zhou M. Todorova G. (2019). Should authentic leaders value power? A study of leaders’ values and perceived value congruence. J. Bus. Ethics 156, 1027–1044. doi: 10.1007/s10551-017-3617-0

[ref97] Roberson Q. M. (2006). Disentangling the meanings of diversity and inclusion in organizations. Group Org. Manag. 31, 212–236. doi: 10.1177/1059601104273064

[ref99] Sawyer R. K. Henriksen D. (2024). Explaining creativity: The science of human innovation. New York: Oxford University Press.

[ref100] Seggewiss B. J. Boeggemann L. M. Straatmann T. Mueller K. Hattrup K. (2019). Do values and value congruence both predict commitment? A refined multi-target, multi-value investigation into a challenged belief. J. Bus. Psychol. 34, 169–187. doi: 10.1007/s10869-018-9534-0

[ref101] Shafique I. Ahmad B. Kalyar M. N. (2020). How ethical leadership influences creativity and organizational innovation: examining the underlying mechanisms. Eur. J. Innov. Manag. 23, 114–133. doi: 10.1108/EJIM-12-2018-0269

[ref103] Stouten J. Van Dijke M. De Cremer D. (2012). Ethical leadership. J. Pers. Psychol. 11, 1–6. doi: 10.1027/1866-5888/a000059

[ref104] Treviño L. K. Butterfield K. D. McCabe D. L. (1998). The ethical context in organizations: influences on employee attitudes and behaviors. Bus. Ethics Q. 8, 447–476. doi: 10.2307/3857431

[ref105] Tu Y. Lu X. (2016). Do ethical leaders give followers the confidence to go the extra mile? The moderating role of intrinsic motivation. J. Bus. Ethics 135, 129–144. doi: 10.1007/s10551-014-2463-6

[ref106] Tu Y. Lu X. Choi J. N. Guo W. (2019). Ethical leadership and team-level creativity: mediation of psychological safety climate and moderation of supervisor support for creativity. J. Bus. Ethics 159, 551–565. doi: 10.1007/s10551-018-3839-9

[ref107] Tyagi V. Hanoch Y. Hall S. D. Runco M. Denham S. L. (2017). The risky side of creativity: domain specific risk taking in creative individuals. Front. Psychol. 8:145. doi: 10.3389/fpsyg.2017.00145, PMID: 28217103 PMC5289983

[ref108] Usman M. Shahzad K. Khan K. (2015). Islamic work ethics (IWE): a review of litrature and directions for future research. J. Islamic Busi. Manag. 219, 1–28.

[ref109] Van Gils S. Van Quaquebeke N. van Knippenberg D. Van Dijke M. De Cremer D. (2015). Ethical leadership and follower organizational deviance: the moderating role of follower moral attentiveness. Leadersh. Q. 26, 190–203. doi: 10.1016/j.leaqua.2014.08.005

[ref110] Van Knippenberg D. Van Knippenberg B. De Cremer D. Hogg M. A. (2004). Leadership, self, and identity: a review and research agenda. Leadersh. Q. 15, 825–856. doi: 10.1016/j.leaqua.2004.09.002

[ref111] Verbeke W. Ouwerkerk C. Peelen E. (1996). Exploring the contextual and individual factors on ethical decision-making of salespeople. J. Bus. Ethics 15, 1175–1187. doi: 10.1007/BF00412816

[ref650] Victor B. Cullen J. B. (1987). “A theory and measure of ethical climate in organizations’’, in Research in corporate social performance and policy. ed. Frederick W. C. (Greenwich, CT: JAI Press), 5171.

[ref112] Victor B. Cullen J. B. (1988). The organizational bases of ethical work climates. Adm. Sci. Q. 33, 101–125. doi: 10.2307/2392857

[ref113] Vitell S. J. Bing M. N. Davison H. K. Ammeter A. P. Garner B. L. Novicevic M. M. (2009). Religiosity and moral identity: the mediating role of self-control. J. Bus. Ethics 88, 601–613. doi: 10.1007/s10551-008-9980-0

[ref114] Walumbwa F. O. Mayer D. M. Wang P. Wang H. Workman K. Christensen A. L. (2011). Linking ethical leadership to employee performance: the roles of leader–member exchange, self-efficacy, and organizational identification. Organ. Behav. Hum. Decis. Process. 115, 204–213. doi: 10.1016/j.obhdp.2010.11.002

[ref115] Williams L. J. McGonagle A. K. (2016). Four research designs and a comprehensive analysis strategy for investigating common method variance with self-report measures using latent variables. J. Bus. Psychol. 31, 339–359. doi: 10.1007/s10869-015-9422-9

[ref116] Xu A. J. Loi R. Ngo H. (2016). Ethical leadership behavior and employee justice perceptions: the mediating role of trust in organization. J. Bus. Ethics 134, 493–504. doi: 10.1007/s10551-014-2457-4

[ref117] Zhang L. Liu Z. Li X. (2023). Impact of co-worker ostracism on organizational citizenship behavior through employee self-identity: the moderating role of ethical leadership. Psychol. Res. Behav. Manag. 16, 3279–3302. doi: 10.2147/PRBM.S415036, PMID: 37614325 PMC10443685

[ref118] Zhou J. Shalley C. E. (2003). Research on employee creativity: A critical review and directions for future research. Research in personnel and human resources management. 165–217. doi: 10.1016/S0742-7301(03)22004-1

[ref119] Zimmerman N. Kuntz J. Wright S. (2025). Understanding belongingness at work: the self in a relational context. Int. J. Organ. Anal. doi: 10.1108/IJOA-10-2024-4880

