# Supplementary material for: Belonging through values: ethical leadership, creativity, and psychological safety with ethical climate as a moderator
Source: Front Psychol. 2025 May 2;16:1559427. doi: 10.3389/fpsyg.2025.1559427 (PMC12082719; doi:10.3389/fpsyg.2025.1559427)
Supplement: Supplementary file 1 [file Supplementary_file_1.docx]

**Appendix A.**

A.1. Ethical leadership manipulation — high ethical leadership

*Your leader lives his personal life in an ethical way. He is a reliable person and asks himself what is the right thing to do before making decisions. Your leader also takes honest and balanced decisions in his work. He listens to what employees have to say and keeps their interest in mind when deciding. At work he discusses the importance of ethical norms and disciplines employees who violate ethical standards. He defines success not only in terms of results, but also in the way the results are obtained. All in all, your leader sets an example of how to do things the right way in terms of ethics.*

A.2. Ethical leadership manipulation — low ethical leadership

*In his personal life, your leader does not care about living life in an ethical way. He is not really a reliable person and rarely asks himself what is the right thing to do before making decisions. In his work, your leader does not always take honest and balanced decisions either. He does not listen to what employees have to say and does not keep their interest in mind when deciding. At work he never discusses the importance of ethical norms and does not pay attention to whether employees behave in accordance with the ethical standards. He defines success only in terms of results, and does not care about the way results are obtained. All in all, your leader is not a good example of how to do things the right way in terms of ethics.*

**Value Congruence Manipulations**

**B.1. High-Value Congruence Condition**

*Imagine working under a leader named Ahmad. You quickly notice that you and Ahmad share similar values and beliefs, both in the workplace and beyond. For instance, Ahmad places a strong emphasis on integrity, aligning with your own belief in doing what is right, even under challenging circumstances. Ahmad also values collaboration and mutual respect, which matches your preference for working harmoniously with others. Furthermore, Ahmad's broader worldview and ethical standards closely reflect your values and the things you prioritize in life.*

**B.2. Low-Value Congruence Condition**

*Imagine working under a leader named Ahmad. From the beginning, you sense a clear misalignment between your values and Ahmad's leadership approach. While you prioritize collaboration and teamwork, Ahmad emphasizes competition and individual success, often rewarding employees who focus solely on goals. Additionally, you notice that Ahmad's ethical outlook and personal priorities differ significantly from your own.*

**C.1. High Ethical Climate Scenario**

*Imagine you are working at a company where the organizational climate strongly emphasizes ethical behavior. The company has clear policies and procedures that promote fairness, transparency, and integrity. For example, during a recent project, the management team ensured that all decisions were made openly and involved input from all relevant stakeholders. The company also has a robust whistleblower policy that protects employees who report unethical behavior. Regular training sessions are conducted to reinforce the importance of ethical conduct, and leaders consistently model ethical behavior in their actions and decisions. This creates an environment where employees feel confident that ethical behavior is valued and rewarded.*

**C.2. Low Ethical Climate Scenario**

*Imagine you are working at a company where the organizational climate does not prioritize ethical behavior. The company lacks clear policies and procedures for handling ethical issues, and decisions are often made behind closed doors without transparency. For instance, during a recent project, the management team bypassed standard procedures to expedite the process, ignoring potential ethical concerns. There is no formal mechanism for reporting unethical behavior, and employees who raise concerns often face retaliation. Training on ethical conduct is infrequent, and leaders frequently engage in behavior that contradicts the company’s stated values. This creates an environment where employees feel that ethical behavior is neither valued nor rewarded.*
